# Comparative Transcriptomic Analysis of the Metabolism of Betalains and Flavonoids in Red Amaranth Hypocotyl under Blue Light and Dark Conditions

**DOI:** 10.3390/molecules28155627

**Published:** 2023-07-25

**Authors:** Shengcai Liu, Xiao Wang, Liyun Peng

**Affiliations:** 1 Institute of Horticultural Biotechnology, Fujian Agriculture and Forestry University, Fuzhou 350002, China; 15216451726@163.com; 2 State Key Laboratory of Conservation and Utilization of Subtropical Agro-Bioresources, College of Life Science and Technology, Guangxi University, Nanning 530005, China; yaeryun454321@163.com

**Keywords:** *Amaranthus tricolor*, blue light, transcriptome, betalains, flavonoids, anthocyanin

## Abstract

Amaranth plants contain abundant betalains and flavonoids. Anthocyanins are important flavonoids; however, they cannot coexist in the same plant with betalains. Blue light influences metabolite synthesis and hypocotyl elongation; accordingly, analyses of its effects on betalain and flavonoid biosynthesis in *Amaranthus tricolor* may provide insight into the distribution of these plant pigments. We analyzed the betalain and flavonoid content and transcriptome profiles in amaranth hypocotyls under blue light and dark conditions. Furthermore, we analyzed the expression patterns of key genes related to betalains and flavonoids. Amaranth hypocotyls were shorter and redder and showed higher betalain and flavonoid content under blue light than in dark conditions. Key genes involved in the synthesis of betalains and flavonoids were upregulated under blue light. The gene encoding DELLA was also upregulated. These results suggest that blue light favors the synthesis of both betalains and flavonoids via the suppression of bioactive gibberellin and the promotion of DELLA protein accumulation, which also suppresses hypocotyl elongation. The metabolite profiles differed between plants under blue light and dark conditions. These findings improve our understanding of the environmental cues and molecular mechanisms underlying pigment variation in *Amaranthus*.

## 1. Introduction

Red amaranth (*Amaranthus tricolor* L.), belonging to the family Amaranthaceae, is the most commonly cultivated leafy vegetable and is widely distributed in warm and tropical regions [1,2,3]. Both the leaves and stems are consumed. The species has a rich profile of essential metabolites, such as betalains, flavonoids, and alkaloids [4,5,6,7,8], with antioxidant, anticancer, antiviral, and antiparasitic effects, as well as protective effects against certain oxidative-stress-related disorders [4,5,6,7,9,10,11,12,13,14,15,16,17,18]. Fresh amaranth often provides substantially greater nutritional value than other vegetables. Furthermore, medical compounds based on amaranth have broad applications in traditional medicine in many countries as part of a complex therapeutic strategy [19,20,21,22]. Accordingly, the nutritional value of amaranth is well established [23].

Aromatic amino acids, including l-tyrosine, l-phenylalanine, and l-tryptophan, are precursors of some important metabolites. l-tyrosine is required for protein biosynthesis and serves as a precursor of various metabolites, such as plastoquinone, tocopherols, rosmarinic acid, isoquinoline alkaloids, catecholamines, and betalains [24,25]. l-phenylalanine and l-tyrosine are precursors of compounds in phenylpropane biosynthesis, and l-tryptophan is a precursor of auxin.

The classic betalain metabolic pathway includes several enzymes, including polyphenol oxidase (PPO), cytochrome P450 (CYP76AD1/5/6), l-dihydroxyphenylalanine 4,5-dioxygenase (DODA), and glucosyltransferase. Some of the pathway steps occur spontaneously to ultimately generate betalains and so on [26,27,28,29,30,31,32,33]. Additionally, betalain synthesis might involve many metabolic pathways, including pathways related to flavonoids [8,34], lignin [25], and alkaloids [35]. 

l-phenylalanine is converted to *trans*-cinnamic acid, caffeic acid, and *p*-coumaric acid, important substances connecting phenylpropane metabolism with flavonoid metabolism. Our previous research has shown that the expression levels of *Chalcone Synthase* (*CHS*) and *Flavanone 3-Hydroxylase* (*F3H*) are higher in red sectors than in green sectors, which might promote flavonoid synthesis, in contrast to *FS* gene expression [8]. However, there is no difference in total flavonoid content between red and green sectors, and this may be explained by the synthesis of other types of flavonoids. For example, reduced betalain accumulation is associated with increased flavonol accumulation in inflorescences in *Bougainvillea* [34]. 

Anthocyanins are the most important types of flavonoids. However, they cannot coexist with betalains in the same plant [36,37]. Betalains are mainly found in Caryophyllales [27,38,39] and some higher fungi [40,41]. However, it is still unclear why the two pigments do not exist within the same plant and why the distribution of betalains is limited to a few species among the Caryophyllales [42]. Based on a comparative genetic study, the transcriptional downregulation of late-acting enzymes might result in a loss of anthocyanins [36]. However, the gene encoding Anthocyanidin Synthase (ANS), a key enzyme in the anthocyanin biosynthesis pathway, has a deletion with respect to the wild-type sequence in species that normally produce anthocyanins, resulting in a lack of anthocyanin synthesis in *Mirabilis jalapa* L. [43]. 

Light is a key environmental factor for various metabolites in plants [44,45,46,47]; it mainly triggers the biosynthesis and accumulation of secondary metabolites [48]. Furthermore, light is one of the most easily controlled environmental factors. Blue light is the most effective wavelength for the synthesis of anthocyanins, flavonoid compounds, betalains, and carotenoids [49,50,51,52,53,54]. However, little is known about the metabolic relation between betalains and flavonoids under blue light and dark conditions in *A. tricolor*.

Therefore, we applied high-throughput sequencing technology to compare samples of amaranth hypocotyls under dark and blue light conditions. Subsequent Gene Ontology (GO) and Kyoto Encyclopedia of Genes and Genomes (KEGG) pathway analyses, as well as quantitative real-time polymerase chain reaction (qRT-PCR) assays, revealed differentially expressed genes (DEGs) related to betalain and flavonoid metabolism. Our results indicated that blue light controls the metabolic relation between betalains and flavonoids, and they provide a reference for studies of the co-existence of betalains and anthocyanins within a plant. Thus, the data presented herein may be useful for the comprehensive characterization of betalain metabolism.

## 2. Results

### 2.1. Determination of Betalain and Flavonoid Content in Amaranth Hypocotyls

**Figure 1:**
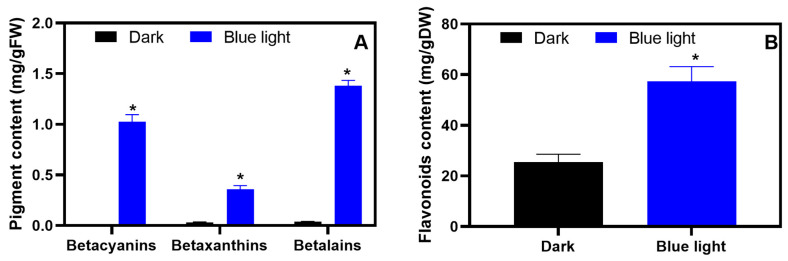
Betalain and flavonoid content in amaranth hypocotyls. (**A**) Betalains; (**B**) flavonoids. * *p* ≤ 0.05.

The amaranth hypocotyls were shorter and redder under blue light than under dark conditions. The betalain and flavonoid content in the amaranth hypocotyls showed a significant difference (*p* ≤ 0.05) between the two conditions (Figure 1), indicating that blue light promoted betalain and flavonoid accumulation in amaranth hypocotyls. 

### 2.2. Transcriptome Assembly for A. tricolor

We extracted RNA separately from the amaranth hypocotyls cultured under blue and dark light to construct cDNA libraries for sequencing. After quality control, an average of 12.71 Gb and 12.63 Gb of high-quality clean reads were obtained in dark samples and blue light samples, respectively. The raw data were deposited in the NCBI Sequence Read Archive database (https://www.ncbi.nlm.nih.gov/sra/SRR5930345 (accessed on 12 August 2017); accession number: SRR5930345). The Q30 percentages (sequencing error rate < 0.1%) for all samples were over 90%. Additionally, the GC content ranged from 42.60% to 43.87%, similar to previous estimates [8].

All high-quality reads were aligned to the *A. hypochondriacus* genome using HISAT and assembled using StringTie. The total mapping ratio ranged from 52.61% to 61.63%, and the unique mapping ratio ranged from 42.67% to 49.29%.

Transcripts with one exon were most abundant, followed by those with two and over 10 exons. The length distribution of transcripts was mainly within 3000 bp. The number of genes with one transcript was highest, followed by genes with two transcripts. The distribution statistics for all transcripts are provided in Supplementary Figure S1.

### 2.3. Alternative Splicing

Alternative splicing (AS) events in dark and blue samples were further classified into types, including alternative transcription start sites (TSS), alternative transcription termination sites (TTS), alternative exon ends (AE), and exon skipping (SKIP), using rMATs tools. For both samples, TSS and TTS were the most frequent AS types, followed by AE, SKIP, IR, and XAE. Global AS regulation in dark samples was similar to that in samples under blue light (Supplementary Table S2 and Figure S2).

### 2.4. Prediction of Novel mRNAs

**Figure 2:**
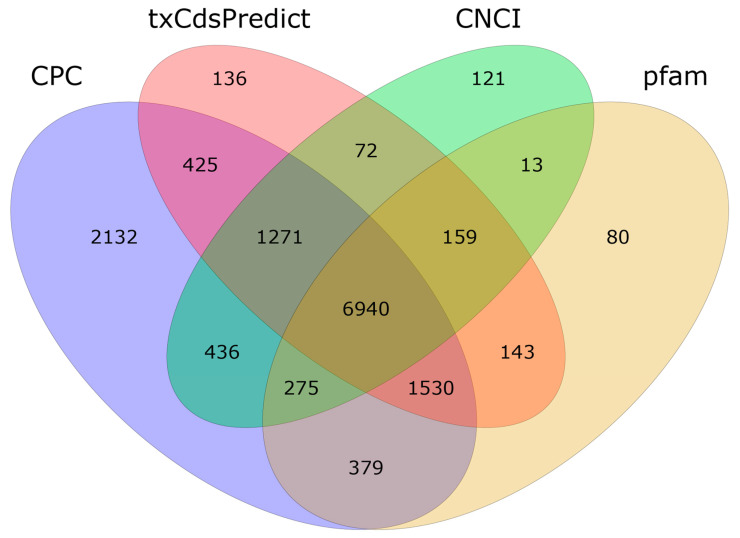
Venn diagram of the prediction results obtained using four databases.

Using CPC ver. 0.9-r2 (threshold > 0), txCdsPredict (threshold > 500), and CNCI (threshold > 0), as well as the Pfam database, we predicted 10,175 novel mRNAs, as shown in Figure 2.

### 2.5. Annotation Analysis of All Protein-Coding Genes

All protein-coding genes were used as queries in a BLASTX search (*E*-value < 1 × 10^−5^) against plant proteins in the Nr, Nt, Swiss-Prot, KEGG, KOG, and GO databases. A total of 34,036 protein-coding genes (97.45%) were obtained (Supplementary Table S3). Among these, 5172 genes were annotated by all databases simultaneously and 33,168 genes were annotated by at least one of the six databases. In particular, 33,023 genes (97.02%) were annotated by the Nr database, 25,710 genes (75.54%) were annotated according to the Swiss-Prot database, 25,710 genes (75.54%) were annotated according to the KEGG database, and 7641 genes (22.45%) were annotated according to the GO database. 

A Venn diagram of the annotation results obtained using the four databases is shown in Supplementary Figure S3. In total, 3837 genes were annotated by the Nr, GO, KEGG, and KOG databases simultaneously and 91,904 genes were annotated by at least one of the four databases.

Based on the KOG annotation, most protein-coding genes (6533) were annotated as ‘General function prediction only’ and 3711 genes were assigned to the ‘Signal transduction mechanism’ classification. Additionally, 2978 and 2790 genes were annotated as ‘Function unknown’ and ‘Posttranslational modification, protein turnover, and chaperones’, respectively. Moreover, 2636 and 1090 genes were annotated as ‘Transcription’ and ‘Secondary metabolite bio-synthesis, transport, and catabolism’. The KOG annotation results are shown in Supplementary Figure S4A.

In the GO classification analysis, 7641 protein-coding genes were assigned to terms in the three main GO categories: biological process, cellular component, and molecular function. In the biological process category, 3116 protein-coding genes were related to cellular processes, while 2871 protein-coding genes were related to metabolic processes. The number of protein-coding genes involved in other processes was fewer than 1000. In the cellular component category, genes related to ‘cell’ (3297) and ‘cell part’ (3252) were overrepresented. The ‘binding’ and ‘catalytic activity’ subcategories were dominant in the molecular function category. The results of the GO classification analysis are shown in Supplementary Figure S4B.

In total, 25,710 genes were assigned to KEGG pathways, including cellar process, environment information process, genetic information process, metabolism, and organismal system pathways. The greatest number of genes were involved in metabolism, including carbohydrate metabolism (2176), amino acid metabolism (1140), and other metabolic pathways (Supplementary Figure S4C).

These protein-coding genes mainly matched homologues in Caryophyllales (Supplementary Figure S4D), such as *Beta vulgaris* (41.33%), *Chenopodium quinoa* (30.11%), and *Spinacia oleracea* (19.57%). *A. tricolor* belongs to Caryophyllales, suggesting that the annotation results were reliable.

### 2.6. Analysis of Differently Expressed Genes Based on FPKM

Gene expression levels were normalized by FPKM. The FPKM values for the known genes and novel genes were 14,019.67 ± 59.34 and 8234 ± 7.55 in samples under dark conditions and 14,204 ± 294.27 and 8238.67 ± 12.34 in samples under blue light, respectively (Supplementary Table S4).

We calculated the transcript abundances of genes based on FPKM. The numbers of genes with different transcript abundances were similar between samples in dark and blue light conditions (Supplementary Figure S5A).

A hierarchical clustering analysis of the screened gene expression levels was performed. The genes with the same or similar expression profiles were clustered (Supplementary Figure S5B,C).

### 2.7. Analysis of DEGs between Dark and Blue Light

**Figure 3:**
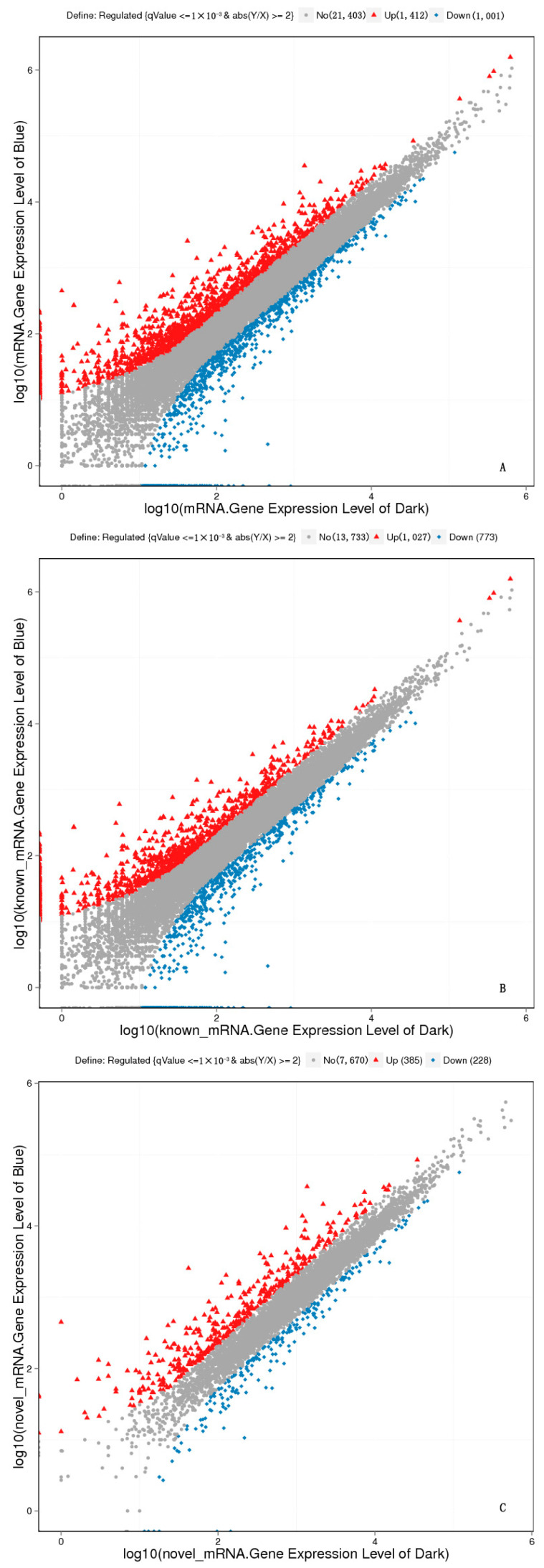
DEGs between samples under dark and blue light conditions. (**A**) Total genes; (**B**) known genes; (**C**) novel genes.

To identify the DEGs between samples in dark and blue light conditions, an FDR set to 0.001 was used as a threshold. Of the 23,816 genes, we identified 2413 DEGs, including 1800 known genes and 613 novel genes. In total, 1412 and 1001 genes were upregulated and downregulated, respectively (Figure 3). These DEGs were distributed on different chromosomes of *A. hypochondriacus*. The largest number of DEGs were located on chromosome 1 (Scaffold_1). A few DEGs were located on an uncertain chromosome (Supplementary Figure S6).

### 2.8. Functional Enrichment Analysis of DEGs

**Figure 4:**
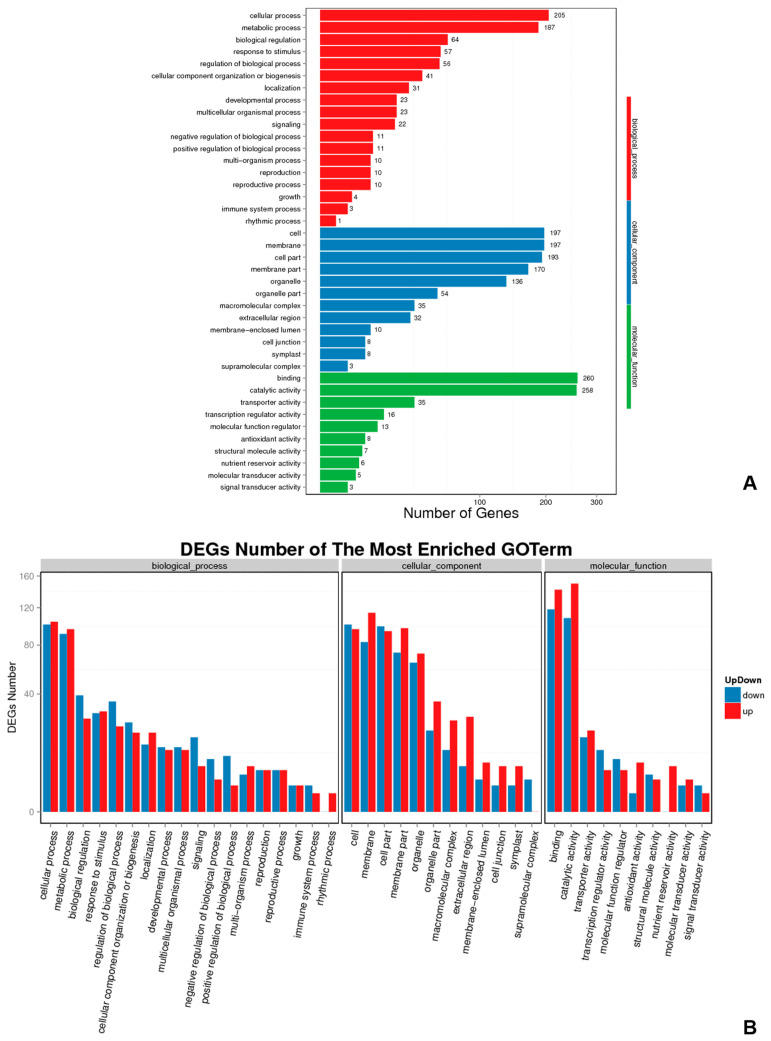
GO classification of differentially expressed genes. (**A**) GO statistics for differentially expressed genes; (**B**) GO statistics for upregulated and downregulated genes.

We performed enrichment and classification analyses of the DEGs by searches against the GO database (Figure 4A). In the biological process category, DEGs were highly enriched for the GO terms cellular process and metabolic process. In the cellular component category, we observed enrichment for cell, membrane, cell part, membrane part, and organelle. Binding and catalytic activity were overrepresented terms for DEGs in the molecular function category. More DEGs related to GO terms in the biological process category were downregulated than upregulated under blue light conditions, and the opposite pattern was observed for DEGs in the cellular component and molecular function categories (Figure 4B).

**Figure 5:**
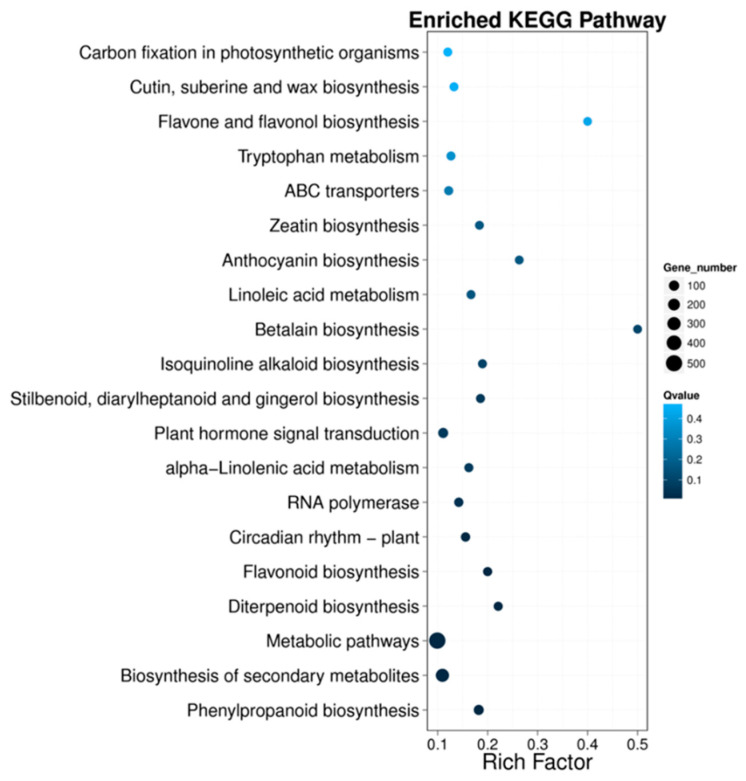
KEGG enrichment analysis of differentially expressed genes.

To explore potential genes regulating the distinct metabolite profiles of *A. tricolor*, we further performed KEGG enrichment analyses of the DEGs between samples under dark and blue light conditions. These DEGs were significantly enriched for several pathways. Phenylpropanoid biosynthesis; biosynthesis of secondary metabolites; metabolic pathways; diterpenoid biosynthesis; flavonoid biosynthesis; circadian rhythm—plant; RNA polymerase; alpha-linolenic acid metabolism; plant hormone signal transduction; stilbenoid, diarylheptanoid, and gingerol biosynthesis; isoquinoline alkaloid biosynthesis; and betalain biosynthesis were the ten most highly pathways (Figure 5).

We found that most genes involved in phenylpropanoid biosynthesis and flavonoid biosynthesis were upregulated under blue light conditions compared to dark conditions. However, genes involved in anthocyanin biosynthesis were downregulated. 

A large number of DEGs were involved in secondary metabolism, verifying that blue light could stimulate secondary metabolism. Furthermore, genes involved in betalain biosynthesis and anthocyanin biosynthesis were both significantly differentially expressed.

### 2.9. qRT-PCR Analysis

**Figure 6:**
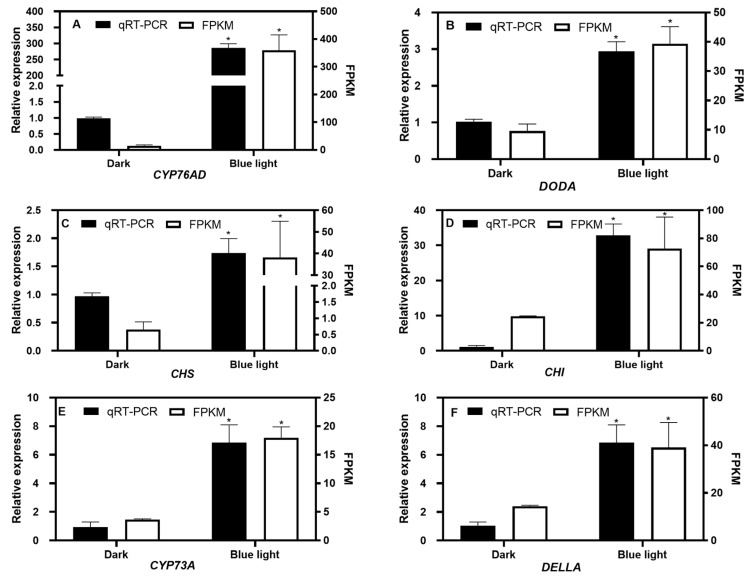
qRT-PCR analysis under the Dark and Blue light condition. * *p* ≤ 0.05. (**A**) *CYP76AD* and (**B**) *DODA* gene were involved in betalain metabolism; (**C**) *CHS*, *(***D***) CHI*, and (**E**) *CYP73A* gene were involved in flavonoid metabolism. (**F**) *DELLA* gene was involved in the gibberellin signaling response.

We detected the expression levels of genes involved in the synthesis of betalains and flavonoids by qRT-PCR (Figure 6). The expression levels of genes related to betalain and flavonoid metabolism, such as *CYP76AD1*, *DODA*, *CHS*, *CHI*, and *CYP73A*, were higher under blue light than under dark conditions. The *DELLA* gene, involved in the gibberellin signaling response, was also upregulated under blue light.

## 3. Discussion

### 3.1. Evaluation of the Amaranth Transcriptome

Illumina HiSeq sequencing is a low-cost approach compared with other sequencing technologies and enables rapid and efficient high-throughput sequencing. RNA sequencing has been widely used to study the regulatory mechanisms underlying the plant response to stress and pigment metabolism [55,56,57,58,59]. We have previously performed de novo transcriptome sequencing to evaluate in vitro plantlet growth and flowering [60] and betalain metabolism in amaranth [8]. The completion of *A. hypochondriacus* genome sequencing [61] provides a reference genome for the transcriptome sequencing of *A. tricolor*. The GC content ranged from 42.60% to 43.87%, which is similar to previous estimates [8]. We obtained 34,036 protein-coding genes, of which 33,168 genes were annotated. These protein-coding genes mainly matched homologues in *Beta vulgaris* (41.33%), *Chenopodium quinoa* (30.11%), and *Spinacia oleracea* (19.57%). *A. tricolor* belongs to Caryophyllales, supporting the reliability of the annotation results.

### 3.2. Blue Light Is Beneficial for Flavonoid and Betalain Accumulation in Amaranth

Among several environmental factors, light is likely the most important determinant of various plant metabolites [44,45,46,47]; in particular, it can trigger the biosynthesis and accumulation of secondary metabolites [48]. Light is also one of the most easily controllable environmental factors. Amongst various wavelengths, blue light is the most effective for the synthesis of anthocyanins, flavonoid compounds, betalains, and carotenoids [49,50,51,52,53,54]. Extensive research indicates that cytochrome P450 (*CYP76AD1/5/6*) and l-dihydroxyphenylalanine 4,5-dioxygenase (*DODA*) are vital enzymes in the betalain metabolic pathway [26,27,28,29,30,31,32,33]. In the study, we found that genes involved in betalain biosynthesis via the tyrosine metabolism pathway were upregulated, including *CYP76AD* and *DODA*. 

A previous study has revealed that betalain accumulation is reduced when flavonol accumulation increases in the inflorescences in *Bougainvillea* with color variation [34]. Furthermore, we speculated that the total flavonoid content would not differ between the red and green sectors of *A. tricolor* because other types of flavonoids might be biosynthesized [8]. Our results showed that blue light could upregulate genes involved in phenylpropane biosynthesis based on the KEGG pathway map (https://www.kegg.jp/kegg/pathway.html) (accessed on 20 February 2017), especially genes related to flavonoid biosynthesis(https://www.kegg.jp/kegg/pathway.html) (accessed on 18 April 2017) (shown in Supplementary Figure S7A,B). These results suggest that blue light is beneficial for flavonoid and betalain accumulation in amaranth, different from seedlings cultured under normal conditions or at different development stages. This effect is more complex regarding blue light’s influence on flavonoid and betalain accumulation in amaranth.

### 3.3. Transcriptional Downregulation of Genes Related to Anthocyanin Synthesis Might Result in Loss of Anthocyanins in A. tricolor

Anthocyanins are critical flavonoids; however, they cannot coexist in the same plant with betalains [36,37]. It is still unclear why anthocyanins and betalains are not found within the same plant and why betalains only exist in a few species among the Caryophyllales [42]. In *Mirabilis jalapa* L., a sequence deletion in *ANS*, which encodes a key enzyme in the anthocyanin biosynthesis pathway, resulted in a lack of anthocyanin synthesis [43]. The transcriptional downregulation of late-acting enzymes might also result in a loss of anthocyanins [36]. Our results showed that the genes involved in anthocyanin synthesis based on the KEGG pathway map (https://www.kegg.jp/kegg/pathway.html) (accessed on 6 June 2013) were downregulated (Supplementary Figure S8), which may result in a loss of anthocyanins in *A. tricolor*.

### 3.4. Blue Light Might Promote DELLA Protein Accumulation for the Regulation of Betalains and Hypocotyl Elongation in Amaranth

GAs are tetracyclic diterpenoid plant hormones that control plant growth, development, and metabolism [62,63,64]. Only a few of the presently known 126 GAs are physiologically active, including GA_1_, GA_3_, GA_4_, and GA_7_ [65]. Light and GAs mediate many important and partially overlapping plant developmental processes. DELLA proteins, as GA signaling repressors, can prevent GA-induced development, and GA promotes the degradation of DELLA proteins to attenuate their suppressive effect on the GA signaling pathway [66,67]. We found that blue light suppressed hypocotyl elongation and promoted betalain synthesis, consistent with our previous results [68,69]. Moreover, GAs negatively regulated flavonoid biosynthesis through GA-mediated signaling pathways in leaves in *Medicago truncatula* [70]. Our analysis of the transcriptome database indicated that genes related to GA12 and GA1 (or GA4) were upregulated and downregulated, respectively. Genes encoding DELLA were upregulated. We speculated that blue light inhibited the levels of bioactive GAs and suppressed the degradation of DELLA proteins by GA induction. These results further support the role of DELLA in the regulation of amaranth seedling growth and betalain and flavonoid biosynthesis via GA signaling.

## 4. Materials and Methods

### 4.1. Materials and Treatment

Amaranth seeds were purchased from Nanjing Jinshengda Seed Co., Ltd. (Nanjing, China). After disinfection treatment with 75% alcohol for 30 s, 0.1% mercuric chloride for 6 min, and finally rinsing with sterile water 5–6 times, they were placed on plastic Petri dishes covered with three layers (Beimu, Hangzhou, China) of filter paper moistened with sterile water for germination under dark and blue light (440–500 nm) conditions at 25 ± 2 °C in a light incubator. 

**Figure 7:**
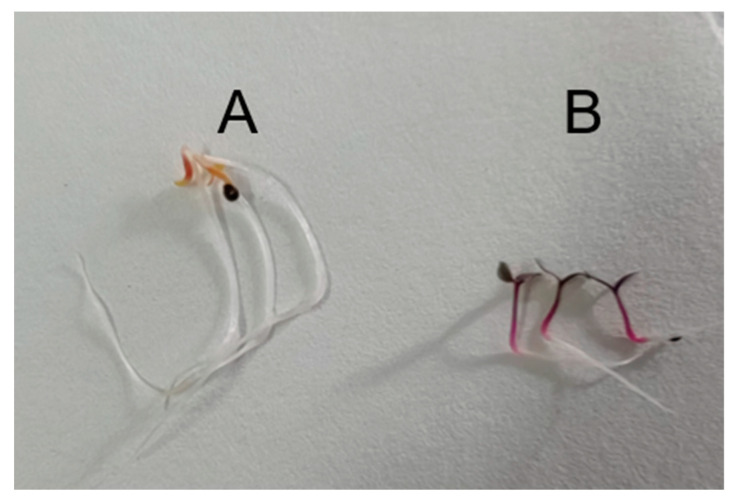
Seed germination of amaranth. (**A**) Seed germination under dark conditions; (**B**) seed germination under blue light conditions.

After 3 days, the fresh amaranth hypocotyl was collected to extract the betalains, as described by Liu [8] (Figure 7). Betacyanins and betaxanthins were detected spectrophotometrically at 538 and 470 nm and quantified by using the molar extinction coefficient 60,000 and 48,000 M^−1^cm^−1^, respectively. The dried samples were collected to determine the flavonoids content, referring to the extraction and determination protocol (Comin Biotechnology Co., Ltd., Suzhou, China). Samples were frozen in liquid nitrogen for RNA extraction. All treatments were performed with three biological repetitions. The data were analyzed by one-way analysis of variance (ANOVA) followed by Duncan’s test with a level of significance of *p* ≤ 0.05 using SPSS version 19.0 (IBM Corp., Armonk, NY, USA).

### 4.2. Total RNA Extraction and Quality Detection

Total RNA was extracted using the CTAB method and treated with DNase I to eliminate residual genomic DNA [71]. The extracted RNA was quantified using a NanoDrop 2000 spectrophotometer (Thermo, Waltham, MA, USA), and its integrity was assessed with an Agilent 2100 Bioanalyzer (Agilent Technologies, Santa Clara, CA, USA) and by denaturing agarose gel electrophoresis with ethidium bromide staining [72]. For each sample, the RNA concentration and amount exceeded 300 ng/μL and 15 μg, respectively.

### 4.3. Construction, Detection, and Sequencing of Transcriptome Libraries

Ribosomal RNA (rRNA) was removed from the total RNA using the Ribo-Zero rRNA Removal Kit (Illumina, San Diego, CA, USA). Subsequently, a cDNA library was constructed for each sample using the TruSeq Stranded Kit according to the manufacturer’s instructions (Illumina). The cDNA library quality and fragment lengths were evaluated using the Agilent 2100 DNA 1000 Kit, after which the cDNA libraries were sequenced using the Illumina HiSeq Xten system to generate 125-bp paired-end reads.

### 4.4. Sequencing Data Assembly

The raw data generated from the sequencing of each cDNA library were transformed into sequence data (i.e., raw data or raw reads) by base calling. Adapter fragments, reads with ambiguous bases (“N”), and reads with more than 10% of bases with a Q-value of <30 were removed to obtain clean reads for subsequent analyses.

Clean reads were mapped to *A. hypochondriacus* genome release version 2.1 (http://www.phytozome.net/) (accessed on 8 May 2017) using HISAT [73], followed by further assembly using StringTie [74]. 

After transcript reconstruction, all transcript sequences in each sample were obtained. These transcripts were compared with *A. hypochondriacus* mRNAs using Cuffcompare [75] to obtain the locations of transcripts on the *A. hypochondriacus* genome. Alternative splicing (AS) events were detected and quantified according to FPKM values for each sample using Asprofile b-1.0.4 (http://ccb.jhu.edu/software/ASprofile/) [76].

Genes with low expression levels may have been incompletely assembled in each replicate owing to an insufficient sequencing depth. Thus, Cuffmerge [75] was used to obtain the complete transcripts of amaranth hypocotyls under dark and blue light conditions.

### 4.5. Prediction of Coding Ability and Annotation Analysis

CPC v0.9-r2 (threshold score > 0) [77], txCdsPredict (threshold > 500) [78], and CNCI (threshold > 0) [78], as well as the pfam database [79], were used to predict protein-coding genes. Transcripts were identified as mRNAs when at least three consistent judgments were obtained. Predicted mRNAs were used for subsequent analyses. 

The coding genes were then used as queries in a BLAST search against the NCBI Nt databases [80] and in searches against the non-redundant (Nr), Swiss-Prot, euKaryotic Orthologous Groups (KOG), and KEGG databases using Diamond ver. 0.8.31 [81]. GO annotation and InterPro annotation were performed using Blast2GO Pipeline ver. 2.5.0 [82] and InterProScan 5.11-51.0 [83], respectively.

### 4.6. Analysis of Expression Levels Based on FPKM

Clean reads for each sample were compared with sequences using Bowtie ver. 2.2.5 [84], and expression levels were calculated using RSEM ver. 1.2.12 [85]. The fragments per kilobase of transcript per million mapped reads (FPKM) values were used as estimates of the expression levels. 

To identify genes with similar expression trends, a clustering analysis of the gene expression levels of each sample was performed using pheatmap ver. 1.0.12 [86]. Genes expressed in all samples and genes expressed in at least one sample were obtained.

### 4.7. Annotation Analysis of Differentially Expressed Genes (DEGs)

After normalizing the gene expression levels, the DEGs between dark and blue light conditions were identified using DEGSeq [87]. A *p*-value of ≤0.001 was applied as the threshold after adjustment for multiple comparisons based on the Benjamini and Hochberg false discovery rate (FDR) method. The unique reads with a fold change (FC) ≥2 and FDR ≤ 0.001 were identified as DEGs. Then, an enrichment analysis of the DEGs between the dark and blue light conditions with FDR ≤ 0.01 was performed using the GO and KEGG databases. MapMan 3.6.0 RC1 [88] was used to compare the DEGs with *A. tricolor* metabolic pathways. 

### 4.8. Quantitative Real-Time Polymerase Chain Reaction Analysis

Six DEGs related to flavonoid metabolism and betalains identified by RNA sequencing (RNA-Seq) were selected for validation by qRT-PCR. Gene-specific primers designed using DNAMAN 6.0 (LynnonBiosoft, San Ramon, CA, USA) were synthesized by Shanghai Bio-engineering Co., Ltd. (Shanghai, China). Details regarding the primers are presented in Supplementary Table S1. The RNA samples used to construct the cDNA libraries were used for the qRT-PCR analysis, which was completed with SYBR Green I Master Mix (Takara, Kusatsu, Japan) and the LightCycler 480 qRT-PCR instrument (Roche, Basel, Switzerland). All samples were analyzed in triplicate, with three biological replicates per sample. The *EF1a* gene was used as an internal reference for the calculation of relative unigene expression levels. Specific details regarding the qRT-PCR methods were described previously [47].

## 5. Conclusions

Blue light favors the synthesis of both betalains and flavonoids via the suppression of bioactive gibberellin and the promotion of DELLA protein accumulation, which also suppresses hypocotyl elongation. The transcriptional downregulation of enzymes involved in anthocyanin synthesis resulted in the loss of these pigments in *A. tricolor*. The metabolite profiles differed between plants under blue light and dark conditions.

## Data Availability

All datasets generated for this study are included in the article/Supplementary Materials.

## Supplementary Materials

The following supporting information can be downloaded at: https://www.mdpi.com/article/10.3390/molecules28155627/s1. Figure S1: The distribution statistics of all the transcripts. Figure S2: Alternative splicing events. Figure S3: Venn graph of the four databases’ annotation results. Figure S4: Annotation analysis of the all coding genes. (A) KOG annotation; (B) GO annotation; (C) KEGG annotation; (D) species distribution. Figure S5: Analysis of differently expressed genes based on FPKM. (A) FPKM; (B) hierarchical clustering of the genes expressed in the all samples simultaneously; (C) hierarchical clustering of the genes expressed in at least one of the six samples. Figure S6: Distribution of DEGs on the *A. hypochondriacus* chromosomes. Scaffold_1 to Scaffold_16 represent chromosome 1 to chromosome 16 of *A. hypochondriacus*. (A) Total genes; (B) known genes; (C) novel genes. Figure S7: KEGG pathway map of phenylpropanoid biosynthesis and flavonoid biosynthesis. (A) Phenylpropanoid biosynthesis (https://www.kegg.jp/kegg/pathway.html) (accessed on 20 February 2017); (B) flavonoid biosynthesis (https://www.kegg.jp/kegg/pathway.html) (accessed on 18 April 2017). Red frames and green frames represent upregulation and downregulation, respectively. Figure S8: KEGG pathway map of anthocyanin biosynthesis (https://www.kegg.jp/kegg/pathway.html) (accessed on 6 June 2013). Table S1: Primers for qRT-PCR. Table S2: Statistics of alternative splicing events. Table S3: Statistical data of the coding gene annotation. Intersection: genes were annotated by the all databases simultaneously. Overall: genes were annotated in at least one of the six databases. Table S4: FPKM numbers of the genes (Mean ± SD).

## Abbreviations

TSS	Alternative transcription start site
TTS	alternative transcription termination site
AE	alternative exon ends
ANS	anthocyanidin synthase
CHS	chalcone synthase
CYP76AD1/5/6	cytochrome P450
DEGs	differentially expressed genes
SKIP	exon skipping
F3H	Flavanone 3-Hydroxylase
GO	Gene Ontology
DODA	l-dihydroxyphenylalanine 4,5-dioxygenase
KEGG	Kyoto Encyclopedia of Genes and Genomes
PPO	polyphenol oxidase
