# Supplementary material for: Comparative Transcriptomic Analysis of the Metabolism of Betalains and Flavonoids in Red Amaranth Hypocotyl under Blue Light and Dark Conditions
Source: Molecules. 2023 Jul 25;28(15):5627. doi: 10.3390/molecules28155627 (PMC10420052; doi:10.3390/molecules28155627)
Supplement: Supplementary file 1 [file molecules-28-05627-s001.zip › molecules-2485957-supplementary.pdf]

molecules

Supplementary Materials Figures

Comparative Transcriptomic Analysis of the Metabolism of Betalains and Flavonoids in Red Amaranth Hypocotyl under Blue Light and Dark Conditions

Shengcai Liu [1,*], Xiao Wang [1] and Liyun Peng [2]

[1] Institute of Horticultural Biotechnology, Fujian Agriculture and Forestry University, Fuzhou 35002, China; 15216451726@163.com
[2] State Key Laboratory of Conservation and Utilization of Subtropical Agro-Bioresources, College of Life Science and Technology, Guangxi University, Nanning 530005, China; yaeryun454321@163.com
* Correspondence: 1215698900@qq.com

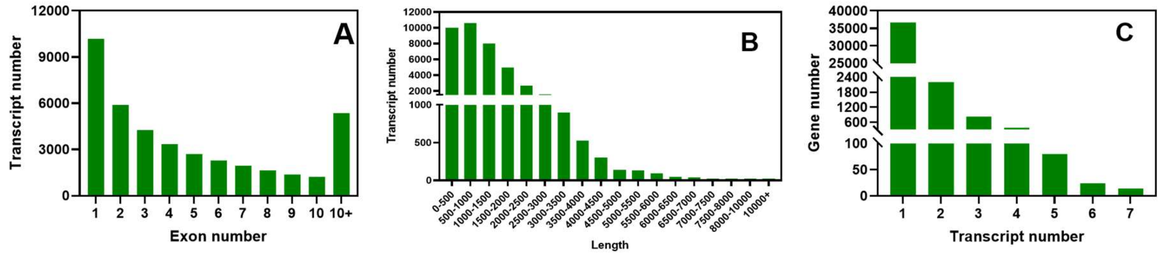

Figure S1: The distribution statistics of all the transcripts. (A): The transcript number with different exon number. (B) The transcript number with different length. (C): The gene number with different transcript number.

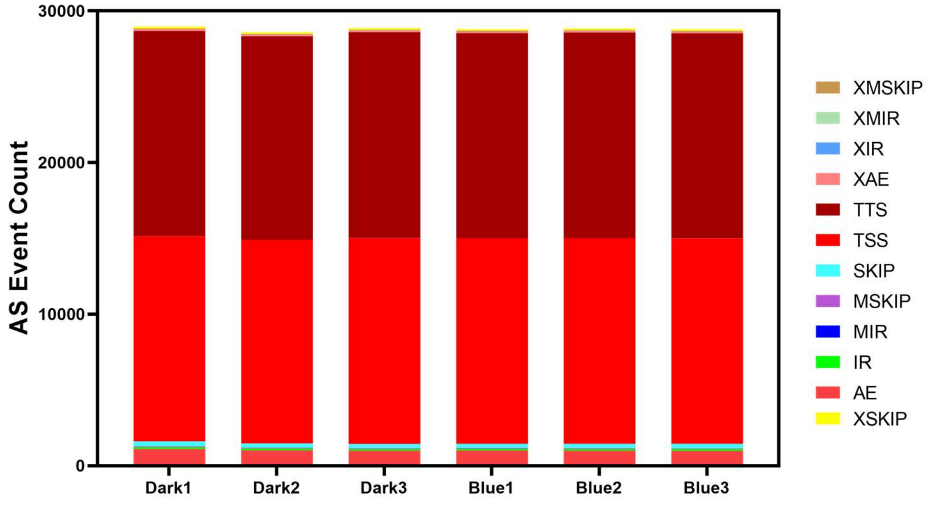

Figure S2: Alternative splicing events.

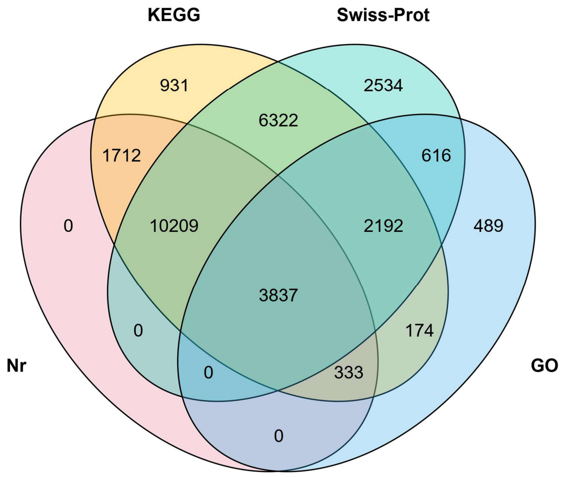

Figure S3: Venn graph of the four databases' annotation results.

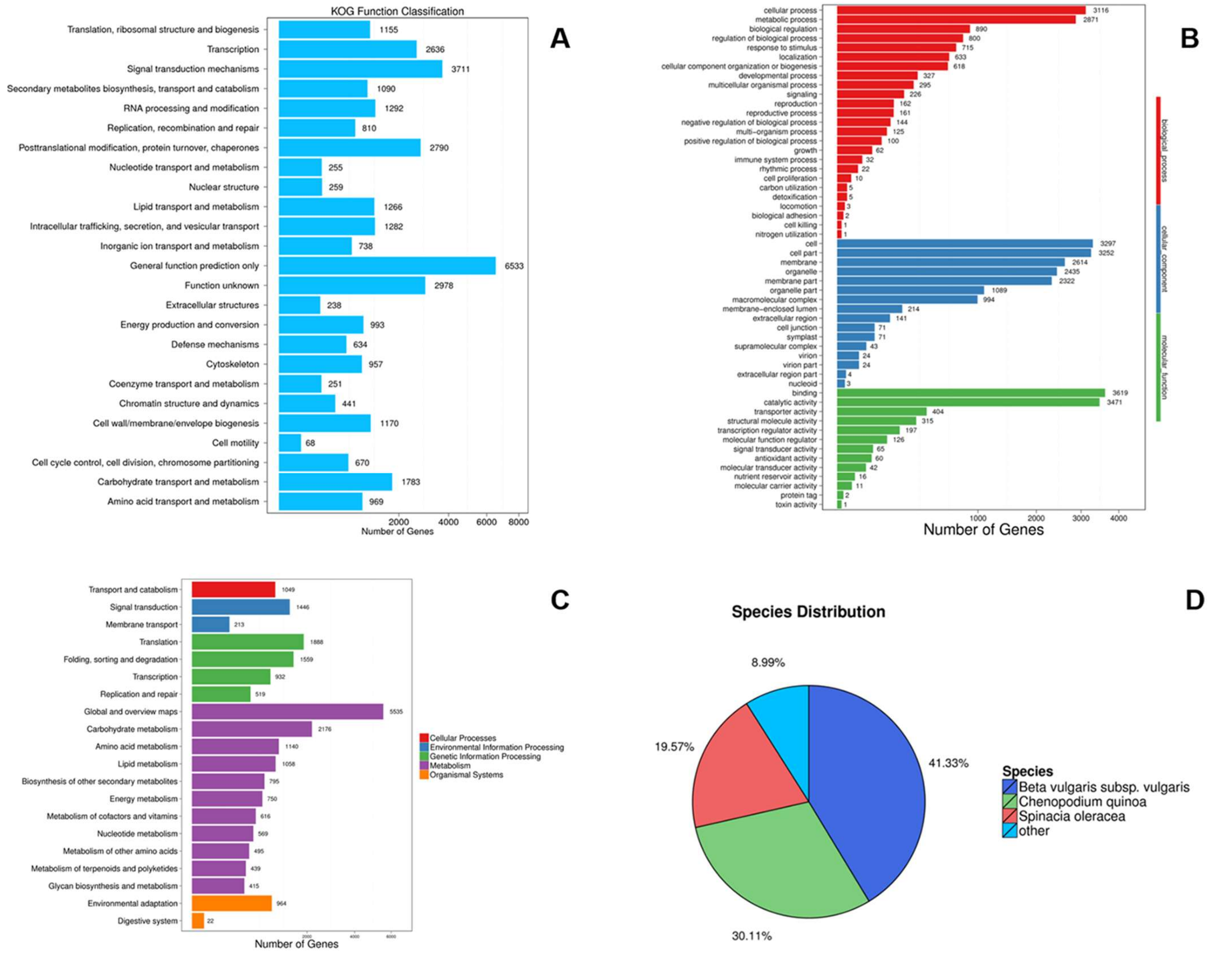

Figure S4: Annotation analysis of the all coding genes. (A) KOG annotation; (B) GO annotation; (C) KEGG annotation; (D) species distribution.

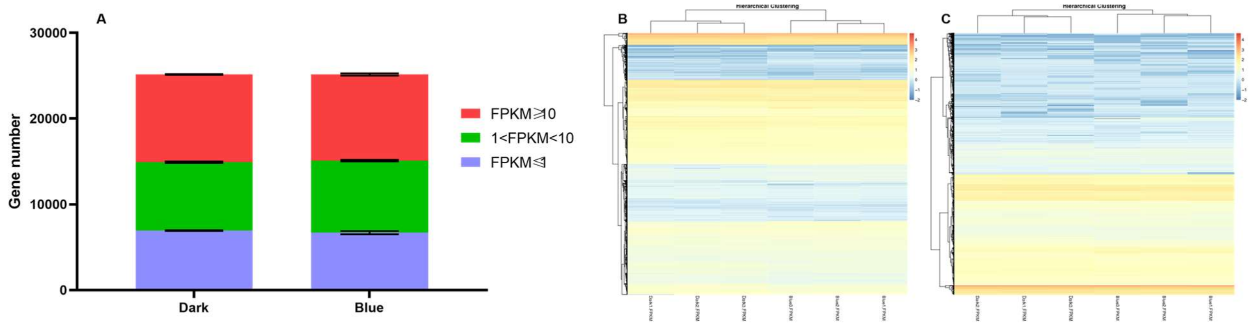

Figure S5: Analysis of differently expressed genes based on FPKM. (A) FPKM; (B) hierarchical clustering of the genes expressed in the all samples simultaneously; (C) hierarchical clustering of the genes expressed in at least one of the six samples.

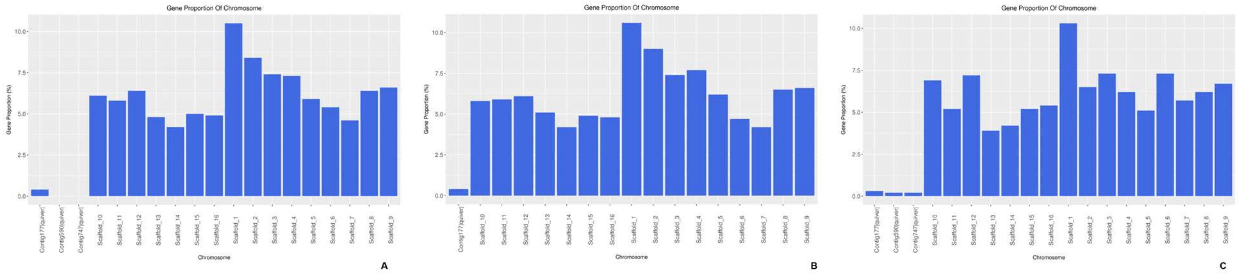

Figure S6: Distribution of DEGs on the *A. hypochondriacus* chromosomes. Scaffold_1 to Scaffold_16 represent chromosome 1 to chromosome 16 of *A. hypochondriacus*. (A) Total genes; (B) known genes; (C) novel genes.

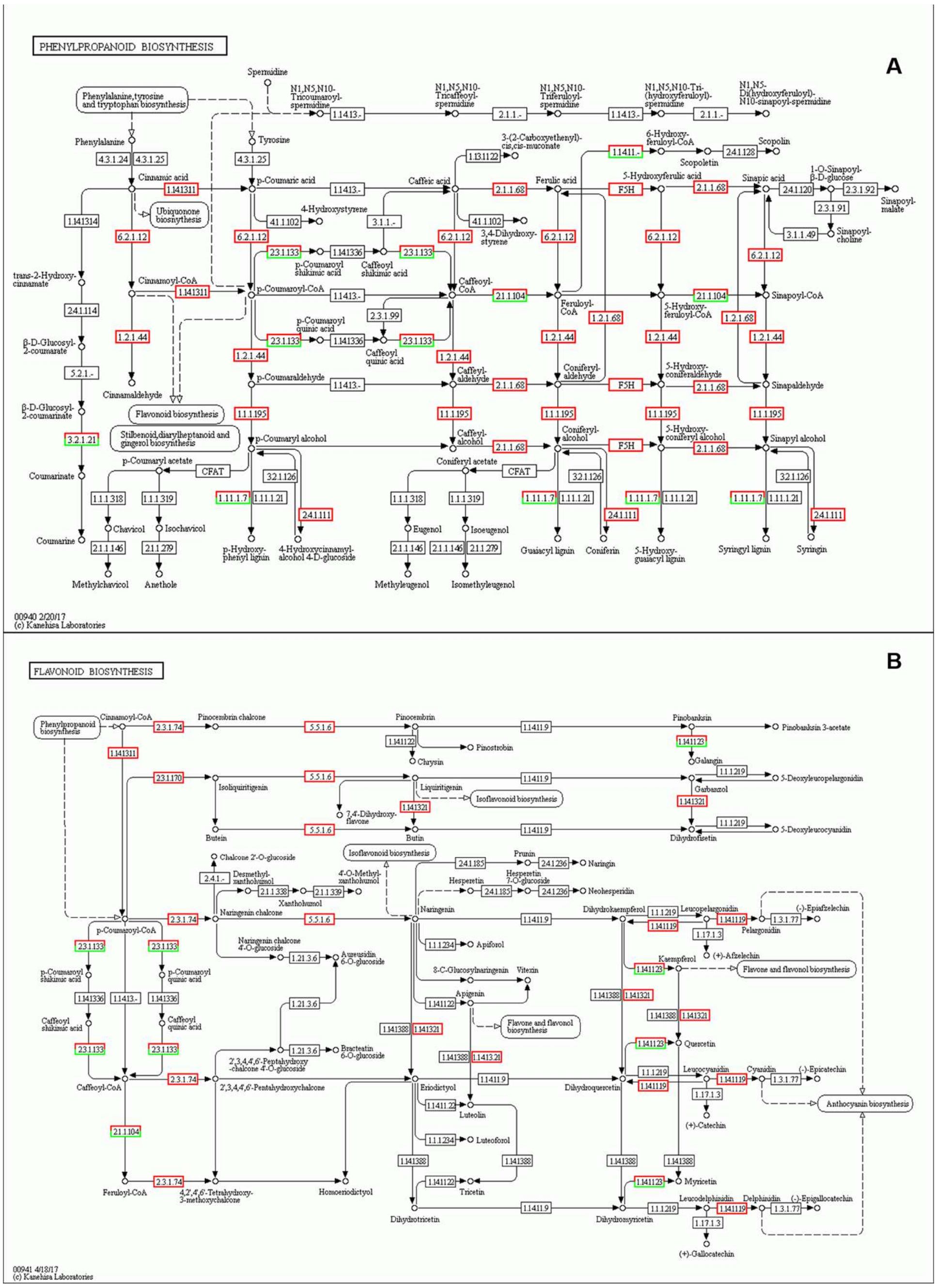

Figure S7: KEGG pathway map of phenylpropanoid biosynthesis and flavonoid biosynthesis. (A) Phenylpropanoid biosynthesis (https://www.kegg.jp/kegg/pathway.html) (accessed on 20 February 2017); (B) flavonoid biosynthesis (https://www.kegg.jp/kegg/pathway.html) (accessed on 18 April 2017). Red frames and green frames represent upregulation and downregulation, respectively.

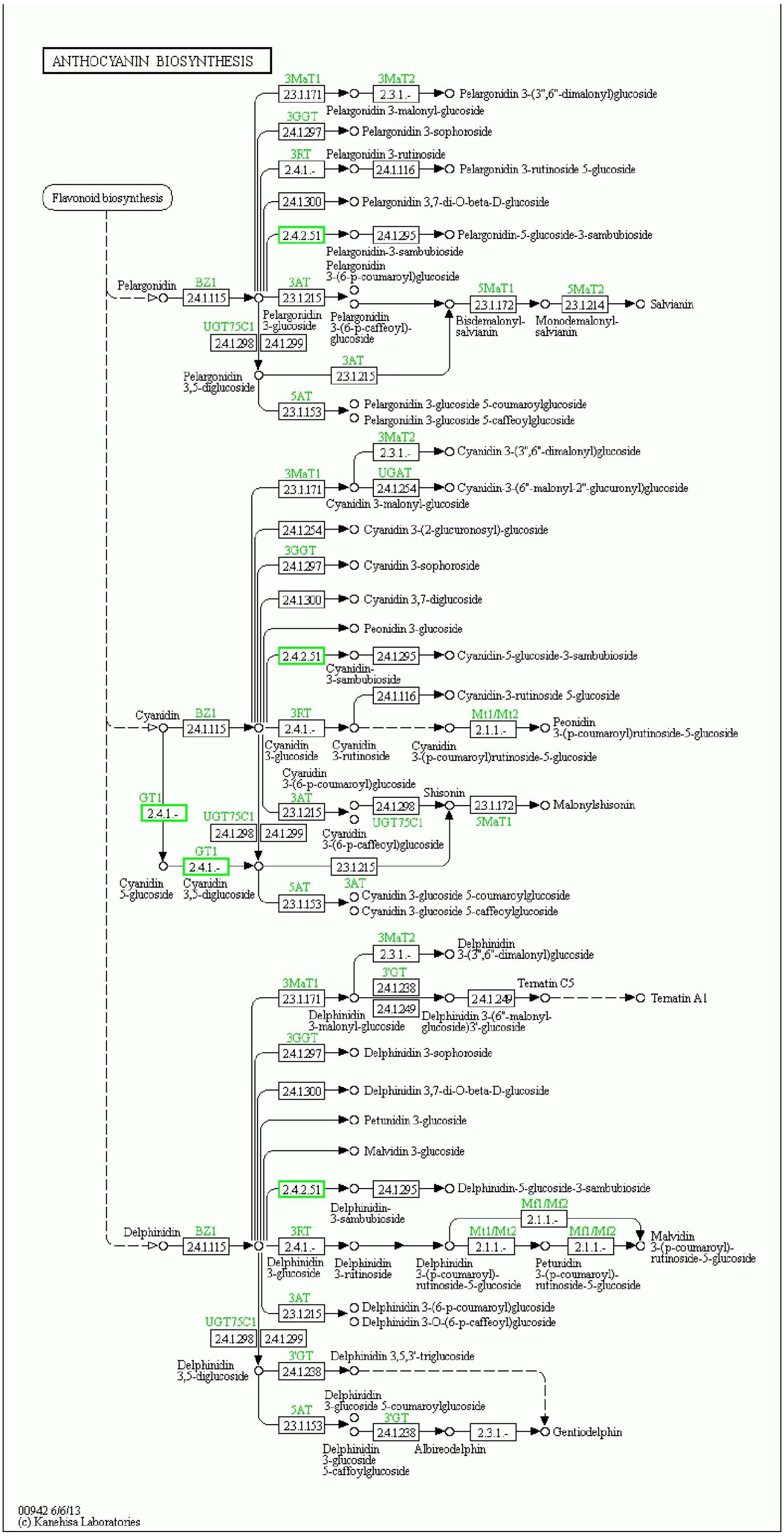

Figure S8: KEGG pathway map of anthocyanin biosynthesis (https://www.kegg.jp/kegg/pathway.html) (accessed on 6 June 2013).
